# The Expected Demand for Elderly Care Services and Anticipated Living Arrangements Among the Oldest Old in China Based on the Andersen Model

**DOI:** 10.3389/fpubh.2021.715586

**Published:** 2021-10-05

**Authors:** Yanbing Zeng, Shuang Que, Chenxi Lin, Ya Fang

**Affiliations:** ^1^School of Public Health, Capital Medical University, Beijing, China; ^2^Key Laboratory of Health Technology Assessment, School of Public Health, Xiamen University, Xiamen, China; ^3^State Key Laboratory of Molecular Vaccinology and Molecular Diagnostics, School of Public Health, Xiamen University, Xiamen, China

**Keywords:** elderly care services, anticipated living arrangement, Andersen model, Chinese, oldest old

## Abstract

**Objective:** The first aim of this study was to explore expected demands of the oldest old and their determinants for different types of elderly care services. The second aim was to investigate preferred choices of living arrangements among the oldest old and the influencing factors.

**Methods:** Data of 4,738 participants aged ≥80 years were extracted from the Chinese Longitudinal Health Longevity Survey carried out in 2014. Using the Andersen model as the analysis framework, a multiple logistic regression analysis was performed to analyze the relationship between the expected elderly care services and living arrangements and other influencing factors. The odds ratios were calculated to indicate the relationship between the influencing factors and the dependent variables.

**Results:** From the descriptive analysis results, we found that the oldest old showed high anticipated needs for home visits (83.5%) and health education (76.4%). Further, there existed a huge imbalance between the supply and demand of care services for the aged. Living with children is still the most important way of providing for the oldest old. The regression results showed that the expected demands for elderly care services and anticipated living arrangements among the oldest old in China are influenced by age, residence, housing property rights, economic status, loneliness, and activities of daily living (ADLs). The oldest old who are older without housing property rights, childless, and have restricted ADLs were more frequently observed to live in long-term care institutions.

**Conclusions:** There is an inequality of the supply and expected demand for elderly care services, and living with children is still a preferred choice of the Chinese oldest old. Our findings indicate that when planning how to promote elderly care services among the oldest old, it is important to consider their expectations, especially for the subgroup that is relatively disadvantaged. Related policies should be developed to offer incentives to family caregivers when they live with the oldest old.

## Introduction

The population of the oldest old (aged ≥80 years) is rapidly growing worldwide (1). In China, the oldest old are assumed to account for 22% of the total population, and the old age support ratio is projected to decline from about 8:1–2:1 by 2050 (2). This situation is among the greatest challenges currently faced by societies, particularly challenging the policymakers. With growing age, the oldest old have a high risk of suffering from health conditions, such as geriatric syndromes, frailty, comorbidities, dementia, and functional decline (3–6). As their functioning worsens, they are most likely cared for by resources from their informal support networks, community services, and government-supported policies or programs.

Given the aging process, how to provide elderly care services has raised urgent concerns in China. The government has proposed that, in the 14th Five-Year period (2021–2025), it is required to develop an elderly care system with high quality. Nevertheless, for rapidly increasing demands for elderly care services, the actual supply of these services has been seriously inadequate. Approximately 60% of older adults needed home visit services, and more than one third needed psychological consulting or daily care services although the proportion of these services provided by the community only accounted for 20% (7). Furthermore, in addition to medical care, there is an upward trend in the diversified needs for rehabilitation, nursing, and spiritual comfort among the older. To promote the development of elderly care services, it is imperative to identify the actual and expected demands of the elderly. That is one of the most important issues we should address as it may further result in more serious health problems and pose higher health burdens on individuals and households. The oldest old is part of the disadvantaged groups that urgently need elderly care services due to their relatively worse physical condition.

In Chinese culture, older adults enjoy a comprehensive status and role in the family through the Confucian norm of filial piety. Living with aging family members and taking care of them is the primary moral principle endorsed by generations of Chinese people. However, with rapid social transitions, including low fertility, increase of urbanization, and individual independence, family size has shrunk on average. According to the seventh national census, the average number of people in each household is fewer than three. Meanwhile, the proportion of the elderly living alone or empty-nest elderly has increased. Family care functions are severely weakened. Although the elderly, as a whole, prefer to live alone (or with spouse only), the preference to live with children was greater in older age groups (8). Therefore, there may be some oldest-old populations who cannot get timely care services. It is required to understand their anticipated living arrangements to provide better support resources.

With respect to care services, previous studies focus on the healthcare service (outpatient and inpatient service) among relatively younger old individuals (9, 10), disabled older people (11), and empty-nest elderly (12). In recent decades, the growth of the older population has increased the demand for elderly care services. Several studies focus on elderly care services among older adults (13, 14). Various factors that influence the demand for these services are taken into consideration and certain contributions made. It is found that gender, career before retirement, family structure, educational attainment, financial strain, and instrumental activities of daily living (ADL) were associated with the need for elderly care services (15, 16). However, the oldest old, at higher demands for elderly care services, remains an understudied and underserved population. Additionally, elderly care services were usually grouped into some categories, such as medical and rehabilitation, instrumental care and support, and psychosocial services, which could limit the purview of these services.

With regard to living arrangements, it has long been regarded as the foundation of elderly care because each household type contained a distinct configuration of demands and resources (17). Previous studies show that living arrangements are critical to health in old age (18, 19). Several factors are suggested to be important in determining the living arrangements of the oldest old, e.g., marital status (20), home ownership (21), and health status (22). However, this research tends to focus on the actual rather than anticipated living arrangement. The actual living arrangement could result from either active or passive acceptance, which may not represent the preferred living arrangement. So far, very little attention has been paid to the role of the preferred living arrangement. Few studies have found that a discrepancy between actual and preferred living arrangements could influence life satisfaction (23) and subjective well-being (24) of older adults. To the best of our knowledge, no study has investigated the influencing factors of anticipated living arrangements among the oldest old. It is important in policy implementation to improve the physical and psychological well-being of the oldest old and social resources allocation.

To address these limitations, this study investigated an extensive range of elderly care services that fit within the Chinese context and anticipated living arrangements among the oldest old. We compared differences between the expected demand and actual supply of various elderly care services for the oldest old. We further identified the factors influencing expected demand for elderly care services and anticipated living arrangements using the Andersen model as a theoretical framework. This study can benefit policymakers in aging countries, such as China, by providing effective and specific policy advice to develop a comprehensive care system.

## Methods

### Data Source

The data was extracted from the seventh wave (2014) of the Chinese Longitudinal Healthy Longevity Survey (CLHLS), which was conducted by the Healthy Ageing and Development Research Center at Peking University, China. It was a high-quality, nationally representative survey, conducted in a random half of the counties and cities in 22 of 31 provinces, covering ~85% of the total population of China (25). The survey adopted a stratified multistage cluster sampling design and was carried out via face-to-face interviews in respondents' homes. It provided rich information on the socioeconomic and demographic characteristics, health-related behaviors and lifestyles, ways of living, and care needs of the population with functional limitations.

From a total of 7,192 individuals, we included 4,738 who were aged ≥80 years and provided answers of expected demand for elderly care services. Subsequently, 549 individuals with missing key data with regard to anticipated living arrangements were excluded from the respective analysis. Therefore, 4,738 and 4,189 oldest-old respondents were included in the analysis on expected demand for elderly care services and anticipated living arrangements, respectively.

The study was approved by the Ethics Committee of Duke University and Peking University (Ethics Number: IRB00001052-13074). Written informed consent was provided by all participants or their legal representatives at baseline and follow-up surveys.

### Dependent Variable

To measure the actual supply and expected demand for elderly care services, participants were asked the following questions in the CLHLS: What kind of social services are available in your community? What kind of social services do you expect to be provided by your community? Their answers with respect to the care services were categorized into the following eight elements of care: (1) daily care services, (2) home visits, (3) psychological consulting, (4) daily shopping, (5) social and recreation activities, (6) legal aid, (7) health education, and (8) neighboring relations. To gather data on anticipated living arrangements, each participant was asked, “Which living arrangement do you prefer?”. One selection was made from a possible four answers: (1) living alone or with spouse no matter how far children live, (2) living alone or with spouse but children living nearby, (3) coresidence with children, or (4) living in a long-term care (LTC) institution.

### Independent Variables

The independent variables in the present study were determined by referring to the Andersen theoretical model. This model was helpful in providing a reasonable scope of factors for investigating the utilization of healthcare services, health-related quality of life, and LTC services (26). It included three groups of factors: predisposing (i.e., age, gender, education, ethnicity, and family), enabling (i.e., financial resources, number of children, and social support network), and need factors (i.e., health status and ADLs) (27). Because this model comprises a variety of factors that may influence the pension system, it can be adopted as an analytical framework to explore problems arising in an aging population. Based on these three domains, we have made conceptual expansions and refinements to make this model more suitable to our research objectives.

The predisposing factors included age (1 = aged 80–89, 2 = aged 90–99, and 3 = aged ≥100), gender (1 = male and 2 = female), residence (1 = urban and 2 = rural), and educational background (1 = no formal education, 2 = elementary school, and 3 = middle school and above). The enabling factors were expressed by housing property rights (1 = own, 2 = rent, and 3 = other), number of children (1 = none, 2 = 1–2, and 3 = ≥3), and economic status (1 = poor, 2 = fair, and 3 = rich). The need factors were measured by self-rated health (1 = bad, 2 = fair, and 3 = good), feeling lonely and isolated (1 = always/often, 2 = sometimes, and 3 = seldom/never), and ADLs (1 = strongly limited, 2 = limited, and 3 = not limited).

### Data Analyses

The analysis started with a description of sample characteristics. We then compared differences in the distribution of elderly care services between expected demand and actual supply and anticipated living arrangements between different age groups. Last, multiple bivariate logistic regressions were used to identify factors that related to every kind of expected demand for elderly care services. To explore the influencing factors on anticipated living arrangements, we performed multivariate logistic regression analysis with “living alone or with spouse no matter how far children live” as our base category. A value of *P* < 0.05 was considered statistically significant. The results are reported as coefficients (β), odds ratios (OR), and 95% confidence intervals (95% CI). Missing data of individual study variables were modest (the highest was 10.6% for educational background), we handled with multiple imputation using the Markov Chain Monte Carlo Simulation. All analyses were conducted in Stata 14.0 for Windows 10.

## Results

### Descriptive Analysis

**Figure 1:**
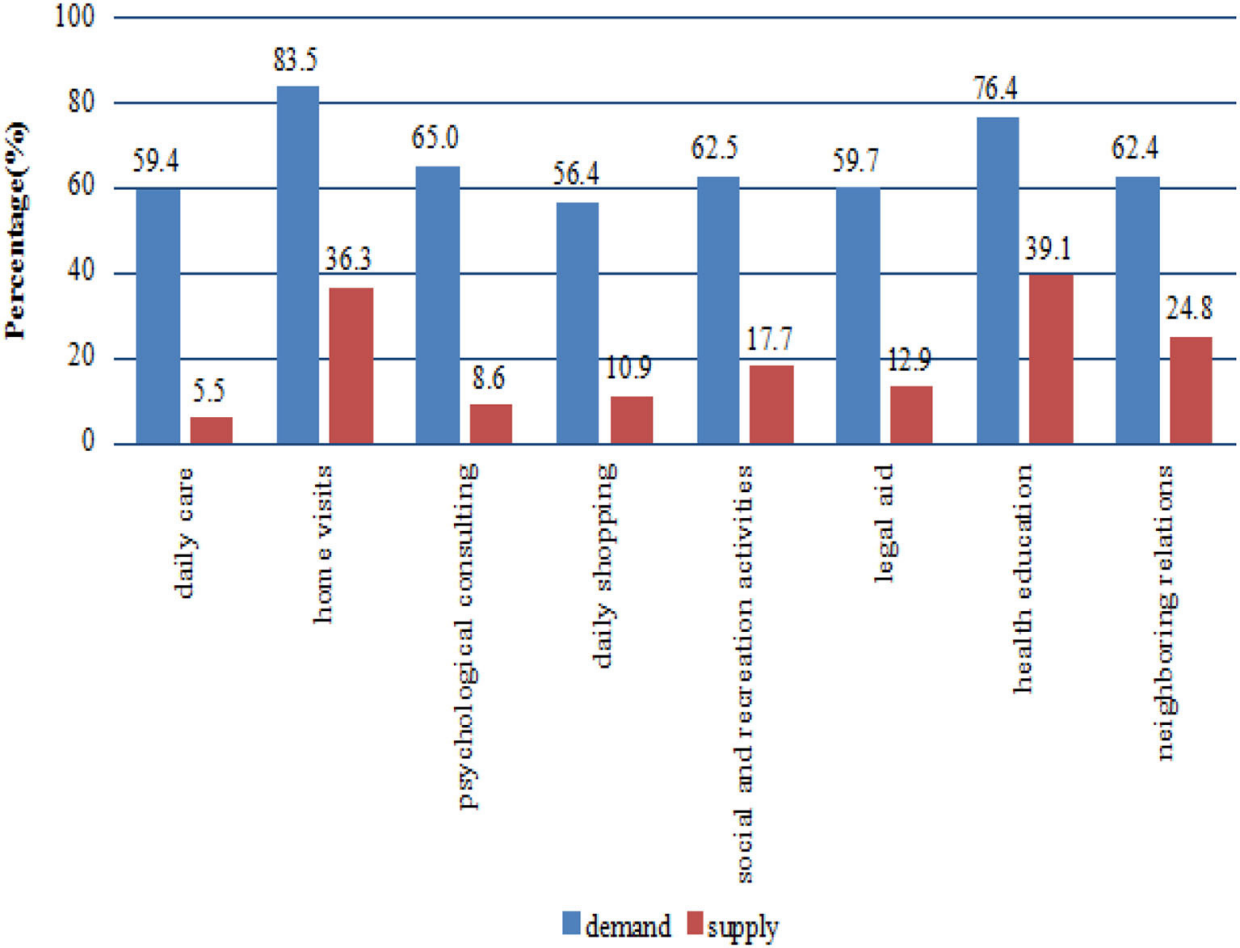
Expected demand and actual supply of various elderly care services for the oldest old.

The main characteristics of the sample are presented in Table 1. Approximately half of the respondents were aged between 80 and 89 years. More than half were female and rural respondents. In addition, approximately two thirds of respondents were illiterate. Figure 1 shows information on expected demand and actual supply of various elderly care services. Generally, relatively fewer services were provided to the participants compared with the demands and did not exceed 40% of the demand. However, a majority of the respondents reported a higher percentage of expected demands being met with regard to elderly care services. The needs for home visit services were the highest, followed by needs for health education, psychological consulting, social and recreation activities, neighboring relations, legal aid, daily care, and daily shopping, in that order. Table 2 presents the anticipated living arrangements among different age groups. As can be seen, the most common anticipated living arrangement was to live with children. Less than 4% of the oldest old chose to go to LTC institutions.

**Table 1:** Descriptive characteristics of the study sample.

**Independent variables**	**Classification**	***N* (%)**
Age (years)	80–89	2,207 (46.6)
	90–99	1,654 (34.9)
	≥100	977 (18.5)
Gender	Male	1,957 (41.3)
	Female	2,781 (58.7)
Residence	Urban	2,074 (43.8)
	Rural	2,664 (56.2)
Years of schooling	0	3,192 (67.4)
	1–6	1,224 (25.8)
	≥7	322 (6.8)

**Table 2:** Anticipated living arrangement categorized based on the age group.

**Dependent variables**	**Age (%)**		
	**Aged 80–89**	**Aged 90–99**	**Aged ≥100**
Living alone or with spouse no matter how far children live	294 (14.4)	95 (6.6)	34 (4.8)
Living alone or with spouse but children living nearby	706 (34.5)	335 (23.3)	126 (17.9)
Co-residence with children	1,002 (49.0)	964 (66.9)	526 (74.8)
Living in an LTC institution	44 (2.2)	46 (3.2)	17 (2.4)

*N = 4,189*.

### Logistic Regression Analysis

The results of the binary regression analyses of expected demand for elderly care services are displayed in Table 3. The oldest old aged 80–89 years had a higher demand for social and recreational activities than the other age groups (OR = 0.784, *P* < 0.01). Participants living in rural areas were in more need of home visit services than those living in urban areas (OR = 1.507, *P* < 0.001). Furthermore, the oldest old without formal education were in greater need for home visits (OR = 0.621, *P* < 0.05) and neighboring relations services (OR = 0.730, *P* < 0.05). In contrast to the oldest old who own a house, those with other housing property rights had an increased need for daily care services (OR = 1.330, *P* < 0.05) and less need for health education (OR = 0.653, *P* < 0.01). Compared with individuals with a low socioeconomic status, those with a rich status needed less daily care services (OR = 0.710, *P* < 0.01). Furthermore, the oldest old with good self-rated health were less likely to report various elderly care service needs except legal aid. The oldest old who sometimes felt lonely and isolated were more inclined to engage in home visits (OR = 1.485, *P* < 0.05), psychological consulting (OR = 1.312, *P* < 0.05), social and recreation activities (OR = 1.471, *P* < 0.01), and health education services (OR = 1.522, *P* < 0.01). Moreover, the oldest old without limited ADLs had a higher demand for social and recreation activities (OR = 1.354, *P* < 0.001), health education (OR = 1.486, *P* < 0.001), and neighboring relations (OR = 1.435, *P* < 0.001) than those with strongly limited ADLs.

**Table 3:** Elderly care service demand for the oldest old (binary logistic regression).

**Independent variables**	**Daily care**		**Home visits**		**Psychological consulting**		**Daily shopping**		**Social and recreation activities**		**Legal aid**		**Health education**		**Neighboring relations**	
	**β**	**OR (95% CI)**	**β**	**OR (95% CI)**	**β**	**OR (95% CI)**	**β**	**OR (95% CI)**	**β**	**OR (95%CI)**	**β**	**OR (95% CI)**	**β**	**OR (95% CI)**	**β**	**OR (95% CI)**
**PREDISPOSING FACTORS**																
**Age (Ref. Aged 80-89)**																
Aged 90–99	0.054	1.056	0.048	1.050	0.043	1.044	−0.025	0.975	−0.161	0.851^*^	−0.129	0.879	−0.047	0.954	−0.134	0.874
		(0.925, 1.206)		(0.879, 1.253)		(0.911, 1.197)		(0.855, 1.112)		(0.744, 0.974)		(0.770, 1.004)		(0.818,1.113)		(0.764,1.001)
Aged ≥100	0.130	1.139	0.040	1.040	0.047	1.048	0.012	1.012	−0.244	0.784^**^	−0.074	0.929	−0.018	0.982	−0.188	0.829^*^
		(0.960, 1.351)		(0.828, 1.308)		(0.880, 1.248)		(0.856, 1.197)		(0.661, 0.929)		(0.784, 1.100)		(0.808, 1.194)		(0.699,0.984)
**Gender (Ref. Male)**																
Female	−0.057	0.944	−0.090	0.914	0.014	1.014	−0.035	0.965	−0.055	0.947	−0.059	0.942	−0.046	0.955	−0.071	0.931
		(0.822, 1.085)		(0.759, 1.100)		(0.880, 1.169)		(0.842, 1.107)		(0.822, 1.090)		(0.821, 1.082)		(0.813, 1.122)		(0.809, 1.072)
**Residence (Ref. Urban)**																
Rural	−0.012	0.988	0.410	1.507^***^	−0.052	0.950	0.062	1.064	−0.063	0.939	−0.057	0.944	0.022	1.022	−0.034	0.967
		(0.874, 1.117)		(1.282, 1.772)		(0.837, 1.077)		(0.943, 1.201)		(0.829, 1.063)		(0.836, 1.067)		(0.887, 1.178)		(0.854, 1.094)
**Years of schooling (Ref. 0)**																
1–6	−0.022	0.979	−0.145	0.865	−0.011	0.989	0.049	1.050	0.035	1.035	0.025	1.026	0.035	1.036	−0.094	0.910
		(0.838, 1.143)		(0.704, 1.062)		(0.844, 1.160)		(0.901, 1.224)		(0.884, 1.213)		(0.878, 1.198)		(0.864, 1.241)		(0.777, 1.065)
≥7	−0.061	0.941	−0.476	0.621^**^	−0.110	0.896	−0.105	0.900	−0.178	0.837	−0.181	0.835	−0.234	0.792	−0.314	0.730^*^
		(0.731, 1.210)		(0.460, 0.839)		(0.693, 1.158)		(0.702, 1.154)		(0.649, 1.079)		(0.650, 1.072)		(0.597, 1.050)		(0.567, 0.940)
**ENABLING FACTORS**																
**Housing property rights (Ref. Owned)**																
Rented	−0.105	0.900	−0.237	0.789	−0.081	0.922	−0.248	0.780	−0.059	0.943	−0.271	0.763	−0.164	0.849	−0.237	0.789
		(0.635, 1.277)		(0.518, 1.203)		(0.645, 1.319)		(0.552, 1.101)		(0.662, 1.343)		(0.540, 1.078)		(0.575, 1.254)		(0.557, 1.119)
Others	0.285	1.330^*^	−0.237	0.789	0.138	1.147	0.186	1.205	0.167	1.181	0.006	1.006	−0.426	0.653^**^	−0.049	0.952
		(1.001, 1.768)		(0.564, 1.104)		(0.860, 1.531)		(0.916, 1.585)		(0.889, 1.569)		(0.765, 1.324)		(0.488, 0.875)		(0.722, 1.256)
**Number of children** **(Ref. 0)**																
1–2	−0.214	0.808	−0.445	0.641	−0.047	0.954	−0.004	0.996	0.059	1.060	−0.073	0.929	−0.141	0.869	0.018	1.018
		(0.537, 1.214)		(0.352, 1.167)		(0.633, 1.436)		(0.675, 1.471)		(0.712, 1.579)		(0.625, 1.383)		(0.543, 1.390)		(0.681, 1.521)
≥3	−0.291	0.747	−0.431	0.650	−0.131	0.877	−0.134	0.875	−0.094	0.910	−0.244	0.783	−0.238	0.788	−0.157	0.854
		(0.510, 1.096)		(0.367, 1.150)		(0.599, 1.285)		(0.608, 1.259)		(0.628, 1.318)		(0.541, 1.135)		(0.508, 1.223)		(0.588, 1.242)
**Economic status (Ref. Poor)**																
Fair	−0.176	0.838	−0.179	0.836	0.083	1.087	−0.052	0.949	0.188	1.207	0.108	1.114	−0.037	0.963	0.086	1.090
		(0.689,1.020)		(0.636,1.099)		(0.893,1.322)		(0.785,1.146)		(0.998,1.460)		(0.922,1.346)		(0.772,1.203)		(0.899,1.322)
Rich	−0.343	0.710^**^	−0.299	0.742	0.025	1.026	−0.151	0.860	0.175	1.192	0.016	1.016	0.107	1.113	0.037	1.037
		(0.559, 0.902)		(0.537, 1.026)		(0.804, 1.308)		(0.680, 1.087)		(0.939, 1.512)		(0.803, 1.286)		(0.843, 1.470)		(0.817, 1.318)
**NEED FACTORS**																
**Self–rated health (Ref. Bad)**																
Fair	−0.157	0.855	−0.085	0.918	−0.086	0.917	−0.066	0.936	−0.048	0.954	−0.024	0.976	−0.068	0.934	0.020	1.021
		(0.712, 1.027)		(0.716, 1.179)		(0.760, 1.107)		(0.783, 1.119)		(0.794, 1.144)		(0.815, 1.168)		(0.758, 1.152)		(0.850, 1.225)
Good	−0.334	0.716^***^	−0.308	0.735^*^	−0.244	0.783^*^	−0.272	0.762^**^	−0.210	0.810^*^	−0.157	0.854	−0.250	0.779^*^	−0.202	0.817^*^
		(0.590, 0.869)		(0.566, 0.954)		(0.642, 0.955)		(0.630, 0.921)		(0.667, 0.984)		(0.706, 1.034)		(0.624, 0.972)		(0.674, 0.992)
**Feeling lonely and isolated (Ref. Always/Often)**																
Sometimes	0.118	1.125	0.395	1.485^*^	0.272	1.312^*^	0.205	1.228	0.386	1.471^**^	0.232	1.262	0.420	1.522^**^	0.200	1.222
		(0.885, 1.430)		(1.092, 2.020)		(1.028, 1.675)		(0.973, 1.550)		(1.162, 1.861)		(0.998, 1.595)		(1.168, 1.984)		(0.962, 1.552)
Seldom/Never	−0.149	0.862	0.252	1.286	−0.068	0.934	0.030	1.031	0.184	1.202	0.027	1.028	0.161	1.174	−0.058	0.944
		(0.685,1.083)		(0.963,1.717)		(0.741,1.177)		(0.825,1.288)		(0.961,1.504)		(0.822,1.285)		(0.915,1.507)		(0.752,1.186)
**ADLs (Ref. Strongly limited)**																
Limited	0.186	1.205	0.229	1.257	0.184	1.201	0.165	1.179	0.162	1.176	0.124	1.132	0.188	1.206	0.187	1.205
		(0.997, 1.456)		(0.980, 1.613)		(0.990, 1.458)		(0.979, 1.420)		(0.974, 1.420)		(0.939, 1.365)		(0.978, 1.488)		(0.999, 1.455)
Not limited	0.163	1.177	0.204	1.227	0.190	1.209^*^	0.242	1.273^**^	0.303	1.354^***^	0.216	1.241^*^	0.396	1.486^***^	0.361	1.435^***^
		(0.984, 1.409)		(0.970, 1.552)		(1.006, 1.453)		(1.067, 1.520)		(1.131, 1.622)		(1.038, 1.484)		(1.214, 1.819)		(1.199, 1.718)

*N = 4,738. Binary logistic regression was adopted. β represents partial regression coefficient; 95% confidence intervals are shown in brackets*.

* *p < 0.05*,

** *p < 0.01*,

*** *p < 0.001*.

The results of the multivariate regression analyses of factors related to the anticipated living arrangement are shown in Table 4. Compared with individuals aged 80–89 years, those aged 100 or more were more likely to be living with their children (OR = 3.206, *P* < 0.001) or living in LTC institutions (OR = 2.815, *P* < 0.001). Females were more inclined to coreside with their children than males (OR = 1.907, *P* < 0.001). The oldest old who completed junior high school or above were less likely to report “living alone or with spouse but children living nearby” (OR = 0.643, *P* < 0.05) and “coresidence with children” (OR = 0.664, *P* < 0.05) compared with those without formal education. With regard to housing property rights, those who did not own or rent a house more often tended to live in LTC institutions (OR = 16.012, *P* < 0.001). Compared with the oldest old without children, those who had three or more children were less likely to live in LTC institutions (OR = 0.082, *P* < 0.001). The oldest old without limited ADLs were less likely to live alone or with spouse but with children living nearby (OR = 0.493, *P* < 0.001) or live with children (OR = 0.385, *P* < 0.001).

**Table 4:** Anticipated living arrangement for the oldest old (multivariate logistic regression).

**Independent variables**	**Living alone or with spouse but children living nearby**		**Co–residence with children**		**Living in LTC institutions**	
	**β**	**OR (95% CI)**	**β**	**OR (95% CI)**	**β**	**OR (95% CI)**
**PREDISPOSING FACTORS**						
**Age** **(Ref. Aged 80–89)**						
Aged 90–99	0.320	1.377^*^ (1.052, 1.801)	1.007	2.738^***^ (2.128, 3.524)	1.222	3.394^***^ (2.004, 5.749)
Aged ≥100	0.232	1.261 (0.832, 1.910)	1.165	3.206^***^ (2.185, 4.705)	1.035	2.815^**^ (1.288, 6.153)
**Gender** **(Ref. Male)**						
Female	0.192	1.211 (0.934, 1.571)	0.646	1.907^***^ (1.491, 2.440)	−0.194	0.824 (0.475, 1.428)
**Residence** **(Ref. Urban)**						
Rural	0.020	1.020 (0.807, 1.289)	−0.072	0.931 (0.746, 1.162)	−0.158	0.854 (0.514, 1.419)
**Years of schooling** **(Ref. 0)**						
1–6	−0.163	0.850 (0.645, 1.120)	−0.254	0.776 (0.596, 1.009)	−0.138	0.871 (0.484,1.565)
≥7	−0.442	0.643^*^ (0.421, 0.981)	−0.409	0.664^*^ (0.446, 0.989)	−0.498	0.608 (0.238, 1.555)
**ENABLING FACTORS**						
**Housing property rights** **(Ref. Owned)**						
Rented	−0.413	0.662 (0.379, 1.156)	−0.687	0.503^*^ (0.296, 0.856)	0.359	1.432 (0.487, 4.212)
Others	−0.195	0.823 (0.469, 1.444)	−0.396	0.673 (0.394, 1.149)	2.773	16.012^***^ (8.302, 30.882)
**Number of children** **(Ref. 0)**						
1–2	0.210	1.234 (0.493, 3.087)	−0.079	0.924 (0.409, 2.090)	−2.125	0.119^***^ (0.043, 0.333)
≥3	0.474	1.606 (0.670, 3.851)	0.038	1.038 (0.478, 2.255)	−2.503	0.082^***^ (0.033, 0.202)
**Economic status** **(Ref. Poor)**						
Fair	0.285	1.330 (0.911, 1.943)	0.100	1.105 (0.777, 1.571)	−0.659	0.517^*^ (0.273, 0.982)
Rich	0.345	1.412 (0.888, 2.245)	0.378	1.459 (0.948, 2.248)	−0.358	0.699 (0.292, 1.674)
**NEED FACTORS**						
**Self-rated health** **(Ref. Bad)**						
Fair	0.361	1.434 (0.989, 2.081)	0.080	1.083 (0.766, 1.533)	−0.218	0.804 (0.415, 1.558)
Good	0.228	1.256 (0.855, 1.845)	−0.077	0.926 (0.647, 1.325)	−0.455	0.635 (0.307, 1.310)
**Feel lonely and isolated** **(Ref. Always/Often)**						
Sometimes	0.332	1.394 (0.873, 2.225)	0.232	1.261 (0.811, 1.961)	0.004	1.004 (0.438, 2.301)
Seldom/Never	0.007	1.007 (0.650, 1.560)	0.127	1.135 (0.753, 1.712)	−0.304	0.738 (0.334, 1.629)
**ADLs** **(Ref. Strongly limited)**						
Limited	−0.473	0.623 (0.381, 1.019)	−0.691	0.501^**^ (0.314, 0.798)	−0.283	0.753 (0.338, 1.681)
Not limited	−0.708	0.493^**^ (0.308, 0.788)	−0.955	0.385^***^ (0.247, 0.601)	−0.717	0.488 (0.224, 1.065)

*N = 4,189. Taking “living alone or with spouse no matter how far children live” as reference, multivariate logistic regression analyses were performed. β represents partial regression coefficient; 95% confidence intervals in brackets*;

* *p < 0.05*,

** *p < 0.01*,

*** *p < 0.001*.

## Discussion

With a rapidly aging population in China, the development of health and social care systems and transition of elderly household patterns are hot topics. In this study, more than half of the oldest old report care service expectations in at least one of these areas. However, the eight types of elderly care services that are available actually only account for small percentages. This result suggests that, despite government efforts to develop elderly care services, there is a relatively marked imbalance between the actual supply and expected demand. For the oldest old, the most important needs were home visits and health education services; thus, these should be focused upon when developing elderly care service systems. In addition, coresidence with children is still the main anticipated living arrangement, which is consistent with previous studies (12). It is important to address the question of the capacity of a family to provide care for elderly parents. Furthermore, we followed the Anderson model to explore factors influencing the needs of Chinese oldest adults with regard to elderly care services and anticipated living arrangements. There were some important findings.

### Expected Demand for Elderly Care Services

First, many predisposing factors were analyzed. For example, we support the effect of residence as mentioned by many other studies (28). In rural areas, the oldest old had a greater need for home visit services than in urban areas, which is very consistent with the reality. The limited access to healthcare services, such as lack of safe, reliable, and accessible public transportation systems and affordable alternatives, are some of the challenges that the rural oldest old encounter (29). Thus, it is not surprising that this group in our study seemed to prefer receiving care services at home.

Second, enabling factors play an important role. The oldest old with more difficult economic circumstances had a greater demand for daily care services. As previous studies indicate, a low income may exacerbate the vulnerability of the oldest old (30). One possible explanation for this phenomenon is that this group tends to have worse health-related outcomes, such as a greater number of chronic diseases and depressive symptoms, which needs more daily care services.

Third, with regard to need factors, our results found that the oldest old who sometimes feel lonely and isolated had a higher demand for psychological consulting and social and recreation activity services. Professional help and support networks of friends may prevent psychological problems and promote social and emotional support. A stable social network is the key factor for preventing loneliness in the oldest old despite their age-related limitations, particularly for those who live alone (31). This reminds us to pay attention to these support services of the oldest old.

### Anticipated Living Arrangements

First, among the predisposing factors, age had a significant impact on the anticipated living arrangements of the oldest old. Compared with people aged 80–89, those aged 100 or older were more likely to live with their children or in LTC institutions and were reluctant to live alone. A possible explanation for this may be that as the age increases, health deteriorates; the oldest old who are aged 100 or older are more vulnerable to poor perceived health and chronic diseases (32), accompanied by functional loss (33) and memory disorder (34). Therefore, it is not surprising that they require informal and formal care services provided by their children or professional nursing staff.

Second, in terms of enabling factors, we found that the oldest old who did not own or rent a house tended to live in LTC institutions. Home ownership can potentially affect an aging parent in determining where and with whom he or she lives (21). A plausible explanation could be that this group without a stable place to live is not able to “age in place” like the homeowners. In addition, living in LTC institutions may be a way to lighten the burden of providing formal care services on their children and family.

Third, in terms of need factors, the oldest old without limited ADLs had lesser likelihood of living with children. In other words, when the oldest old experienced high levels of disability in their ADLs, they were more likely to live with their children, consistent with other research results (34). With regard to ADL limitations, the oldest old have more difficulties with basic ADLs, such as bathing, eating, and dressing, among others. Furthermore, a deterioration in ADLs is a sign of intellectual disability or is associated with other age-related medical conditions (2). Thus, moving in with children may serve as a functional alternative to a nursing home for the oldest old (35).

### Study Limitations

The present study has certain limitations. First, this study used a cross-sectional survey. Therefore, the relationship between identified factors and demand for elderly care services and anticipated living arrangements cannot be interpreted as cause and effect. Second, predisposing, enabling, and need factors included in the analysis are not very comprehensive, and there may be some potential influencing factors not found. Third, perceived economic and health status and loneliness were self-reported, which could lead to the possibility of subjective bias.

### Study Implications

Despite these study limitations, the findings have implications for developing elderly care service systems. Considering the widespread service demands found in this study, the Chinese government needs to develop and improve elderly care services to meet the multilevel and diversified service needs of the oldest old and promote the equilibrium of basic elderly care services. First, driven by demand expression, communities should enrich the content of old-age services and increase high-demand old-age services, such as home visits, health education, and spiritual comfort. Second, targeted services should be provided based on different age groups, places of residence, and health status among the oldest old. Third, it is urgent to highlight the management of service quality and help to promote the refinement of the care services.

With regard to the anticipated living arrangements of the oldest old, living with children is still the most preferred living arrangement for the oldest old in contemporary China. That accords with typical Chinese traditional cultural values, especially filial piety. Considering the changes in the family structure, developing policies related to offering incentives encouraging coresidence is critical. For this, the government could reduce the family caregiver's personal income taxes when they live with the oldest old (13). This is also an effective way to provide assistances for caregivers and reduce the burden of formal care support.

## Conclusions

The dramatic increase in numbers of the oldest old is an urgent concern, presenting a major challenge for existing health and social care systems. In this study, based on findings in the Chinese context, we could afford a reference value for other countries, especially those that similarly emphasize home- and community-based care and familial relationships. We observed an imbalance of the supply and expected demand for elderly care services. Supplies for personal daily care and psychological consulting services were far behind the requirements. Diversified demands for elder care services among the oldest-old individuals were related to age, residence, educational attainment, economic status, and self-rated health. That demonstrated greater shared efforts ought to be devoted to providing adequate elderly care services by assessing their specific service needs of the oldest-old population with different characteristics. Moreover, living with children is still a preferred choice. The anticipated living arrangements were influenced by age, gender, housing property rights, and ADLs. Considering the importance of family care, it is critical to develop incentive policy to encourage coresidence.

## Data Availability Statement

The original contributions presented in the study are included in the article/supplementary material, further inquiries can be directed to the corresponding author/s.

## Ethics Statement

The studies involving human participants were reviewed and approved by the Institutional Review Board (IRB) of the Health System of Duke University. The patients/participants provided their written informed consent to participate in this study.

## Author Contributions

YZ and YF conceived and designed the study and supervised the data analysis. YZ, SQ, and CL wrote the paper. SQ and CL performed all statistical analyses. YZ contributed to revising the paper. All authors contributed to the article and approved the submitted version.

## Funding

This work was supported by the National Natural Science Foundations of China (Nos. 71874147 and 81973144). The funders had no role in the study design, data collection and analysis, decision to publish or preparation of the manuscript.

## Conflict of Interest

The authors declare that the research was conducted in the absence of any commercial or financial relationships that could be construed as a potential conflict of interest.

## Publisher's Note

All claims expressed in this article are solely those of the authors and do not necessarily represent those of their affiliated organizations, or those of the publisher, the editors and the reviewers. Any product that may be evaluated in this article, or claim that may be made by its manufacturer, is not guaranteed or endorsed by the publisher.
